# Emerging Q Fever Infection in Türkiye

**DOI:** 10.1155/jotm/1628136

**Published:** 2026-08-21

**Authors:** Hatun Öztürk Çerik, Arzu Altunçekiç Yıldırım, Celali Kurt, Önder Ergönül

**Affiliations:** ^1^ Department of Infectious Diseases and Clinical Microbiology, Ordu University School of Medicine, Ordu, Turkey; ^2^ Department of Infectious Diseases and Clinical Microbiology, Koc University School of Medicine and American Hospital, Istanbul, Turkey

**Keywords:** *Coxiella burnetii*, endocarditis, hepatitis, Q fever, zoonosis

## Abstract

**Objectives:**

Q fever, caused by *Coxiella burnetii*, is an underrecognized zoonotic infection with diverse clinical manifestations. The nonspecific nature and slow progression of Q fever frequently delay diagnosis, sometimes leading to severe or even fatal outcomes. This study describes 32 patients diagnosed with Q fever over an 18‐month period in a previously unreported region, highlighting the emerging awareness of this neglected zoonosis.

**Methods:**

Patients with seropositive *C. burnetii* Phase I/II IgM and/or IgG antibodies between October 2023 and March 2025 (18‐month period) were included. Cases were classified as acute, probable, chronic, or past infection according to established serological and clinical criteria.

**Results:**

Thirty‐two patients were diagnosed during the study period, of whom 22 (68.8%) were male; the median age was 60.5 years (IQR: 48–66). More than half were farmers, and 40.6% reported animal exposure. Based on serological and clinical criteria, 11 patients were diagnosed with acute Q fever, 16 with probable infection, one with chronic infection, and four with past infection. Common manifestations included fever, myalgia, arthralgia, hepatitis, and endocarditis; cardiovascular involvement occurred in 40.6% of patients. Two patients died, and notably, three patients with prosthetic valve endocarditis had a history of care at the same cardiac surgery clinic.

**Conclusions:**

Given its heterogeneous clinical manifestations and variable serological responses, Q fever poses significant diagnostic challenges. This study presents the first and largest human case series of Q fever from the Black Sea region of Türkiye, highlighting the wide clinical spectrum of the disease and the need for enhanced awareness and diagnostic capacity.

## 1. Introduction

Q fever is a zoonotic disease caused by *Coxiella burnetii*, an obligate intracellular Gram‐negative bacterium. *C. burnetii* has a wide range of reservoirs, including domestic mammals, livestock, wild animals, birds, and arthropods such as ticks, and it is distributed worldwide [1]. Humans are primarily infected through the inhalation of aerosols contaminated with feces, milk, birth products, or bodily fluids from infected animals [2]. The infection may manifest as either an acute or chronic illness. Acute Q fever refers to the primary clinical form of infection and typically presents as a self‐limited febrile illness, pneumonia, or hepatitis, whereas chronic Q fever refers to a rarer persistent focalized infection, most commonly involving the endocardium or vascular structures [3]. Approximately 60% of individuals exposed to the bacterium remain asymptomatic; however, in some cases, the clinical course can be severe and potentially fatal [4]. Due to the nonspecific nature of early symptoms, diagnosis is often delayed or missed. Therefore, a high index of clinical suspicion is especially crucial in high‐risk populations.

Serological methods are highly valuable for diagnosing Q fever. Due to structural changes in the lipopolysaccharide (LPS) of the outer membrane, *C. burnetii* exhibits two distinct antigenic phases: Phase I and Phase II. Phase I corresponds to the virulent form encountered in natural infections, whereas Phase II represents a less pathogenic variant typically generated through repeated passages in cell culture. Antibodies targeting these antigens can be detected using enzyme‐linked immunosorbent assay (ELISA) or indirect immunofluorescence assay (IFA). The pattern of the phase‐specific antibody response plays a crucial role in determining the stage of infection [1, 5].

Thresholds for positive antibody titers may vary between countries. In acute Q fever, Phase II IgG titers typically exceed those of Phase I [3, 4]. For diagnostic purposes, Phase II IgG titers ≥ 1:128 and Phase II IgM titers ≥ 1:48 are commonly accepted as indicative of acute infection, while a Phase I IgG titer ≥ 1:800 is suggestive of chronic infection [5, 6]. When a single serological test yields inconclusive results, repeat testing is recommended [7]. A fourfold increase in antibody titers or seroconversion between paired serum samples collected 2–4 weeks apart supports the diagnosis of acute Q fever [5, 6]. However, because seroconversion requires time to develop, it has been noted that a single serum sample demonstrating the presence of IgM or a high Phase II IgG titer (≥ 1:256) may be sufficient to establish a diagnosis of acute Q fever in most cases [2, 3]. Residual IgG antibodies may persist for years or even a lifetime, regardless of the presence or absence of clinical symptoms. Persistently elevated Phase I antibody titers 6 months after treatment should prompt investigation for chronic infection. Nevertheless, cases of Q fever endocarditis have also been reported in patients with relatively low antibody titers [8].

Q fever is also important from a public health and One Health perspective because transmission is not limited to direct animal contact. In rural settings, occupational and environmental exposure related to livestock, contaminated dust, manure, and field work may increase the risk of infection. Epidemiological studies conducted in Türkiye have demonstrated *C. burnetii* seropositivity in both animals and humans indicating regional circulation of the bacterium, supporting the biological plausibility of ongoing local transmission [2]. However, the number of reported Q fever cases in the national literature remains limited. In Ordu Province, where no previous case reports of acute or chronic Q fever had been published, an increase in Q fever cases has been observed since 2023. In this context, investigating Q fever in the Black Sea region is important for improving clinical recognition and understanding possible local exposure patterns. This study aims to present the epidemiological data, clinical characteristics, and laboratory findings of patients with *C. burnetii* seropositivity at Ordu University Training and Research Hospital.

## 2. Methods

### 2.1. Study Design and Population

This study was designed as a retrospective review of routinely collected clinical data and laboratory records at a tertiary care hospital, covering an 18‐month period between October 2023 and March 2025, which was initiated after the emergence of the index cases in our region. The study included consecutive adult patients aged 18 years and older who were evaluated either in the outpatient setting or during hospitalization and had clinical findings compatible with Q fever, including fever, hepatitis, pneumonia, arthritis, vascular infection, or endocarditis. Serum samples from these clinically suspected patients were analyzed at the National Microbiology Reference Laboratory. Patients with serological evidence of *C. burnetii* Phase I and/or Phase II IgM/IgG antibodies were included in the study. Patients with insufficient clinical records or inadequate laboratory data for case classification were excluded.

### 2.2. Serological Analysis and Case Definitions

Antibodies against *C. burnetii* (Phase I IgG and Phase II IgM/IgG) were detected using both ELISA and IFA (Vircell SL, Spain). Case classification was performed according to the National Infectious Diseases Diagnostic Guidelines published by the Turkish Ministry of Health [6]. IFA‐based phase‐specific titers were used as the primary serological method for diagnostic classification. When available, paired or repeated serum samples were evaluated together to assess seroconversion or dynamic changes in antibody titers over time; in cases of discordant or equivocal ELISA and IFA results, final interpretation was based on phase‐specific IFA findings in conjunction with clinical evaluation.

Definite acute Q fever was defined as a Phase II IgG titer ≥ 1:64 accompanied by a simultaneous IgM titer ≥ 1:48 in an early‐phase serum sample, or a fourfold or greater increase in antibody titers between acute‐ and convalescent‐phase samples.

Probable Q fever was defined as a single serum sample showing a Phase II IgG titer ≥ 1:128 by IFA, or the presence of IgM antibodies detected by ELISA.

Chronic Q fever was defined as a Phase I IgG titer ≥ 1:800.

Past Infection was defined as a serologic evidence of previous exposure without evidence of active acute or chronic Q fever: Phase II IgG reactivity below the study threshold for probable acute infection, absence of Phase II IgM, no documented seroconversion or ≥ 4‐fold rise on follow‐up when available, no compatible acute clinical syndrome, and Phase I IgG below the threshold used for chronic Q fever.

### 2.3. Data Collection

Clinical, demographic, epidemiological, and laboratory data were retrospectively collected from patient files and hospital electronic medical records. Demographic variables included age and sex, while exposure variables included farming, animal contact, tick exposure, and rural residence, when available. Laboratory parameters—including leukocyte, neutrophil, lymphocyte, hemoglobin, and platelet counts; liver function tests (AST, ALT, GGT, ALP); C‐reactive protein (CRP); and erythrocyte sedimentation rate (ESR)—were systematically recorded.

### 2.4. Statistical Analysis

All statistical analyses were conducted using the SPSS 22.0 software package for Windows (SPSS Inc., Chicago, IL, USA). The Kolmogorov–Smirnov test was used to assess the normality of data distribution. Continuous variables were expressed as medians and interquartile ranges (IQR, 25th–75th percentiles), and categorical variables were reported as frequencies and percentages (n, %). This study was performed in line with the principles of the Declaration of Helsinki. The Ethics Committee for Non‐Interventional Scientific Research at Ordu University approved the study (11.04.2025/129).

## 3. Results

During the 18‐month study period, 107 patients underwent serological testing for *C. burnetii* as part of an etiological investigation for distinct infectious and inflammatory syndromes, including fever of unknown origin, atypical pneumonia, acute hepatitis, suspected infective endocarditis, and vascular/graft infections. Among this screened population, 75 patients (70.1%) were found to be seronegative, whereas 32 patients (29.9%) met the study criteria for *C. burnetii* seropositivity and were included in the final analysis.

Of the 32 seropositive patients, 22 (68.8%) were male, with a median age of 60.5 years (IQR: 48–66). Eighteen patients (56.3%) were engaged in farming, 13 (40.6%) reported contact with animals, and one patient had a history of tick exposure. Nineteen patients (59.4%) resided in rural areas; however, no clustering was observed within a specific rural locality, district, or family group.

Based on serological and clinical evaluations, 11 patients were classified as having confirmed acute Q fever, 16 as probable infection, one as chronic infection, and four as past infection. Among those with confirmed acute Q fever, four presented with hepatitis, three with endocarditis, and two with arthritis.

The most commonly reported symptoms among the patients were fever (62.5%), myalgia (68.8%), arthralgia (50.0%), and chronic fatigue (34.4%). Arthritis was observed in 6 patients (18.8%), among whom one also had endocarditis and another had splenomegaly. One patient had HIV coinfection, and three patients had either a history of or concurrent brucellosis. Clinical manifestations among patients with probable infection included fever, myalgia, arthralgia, bursitis, pneumonia, myocarditis, endocarditis, and transverse myelitis. The patient with chronic infection had an abdominal aortic aneurysm graft infection along with concurrent urinary tract tuberculosis. Cardiovascular involvement was identified in 13 patients (40.6%), and two patients died during follow‐up. Notably, three patients with prosthetic valve endocarditis had recently undergone surgery in the same cardiovascular surgery unit. The clinical and laboratory characteristics of the patients are summarized in Tables 1 and 2.

**TABLE 1:** Clinical and laboratory characteristics of patients diagnosed with acute and chronic Q fever.

Patient	Gender	Age (years)	Clinical presentation	Phase I IgG (titer)	Phase II IgM (titer)	Phase II IgG (titer)	Diagnosis
1.	M	65	Hepatitis	1/4096	1/768	1/6384	Acute infection
2.	M	44	Hepatitis	0	1/3072	1/1024	Acute infection
3.	F	86	Hepatitis	1/1024	0	1/16,384	Acute infection (4‐fold rise)
4.	M	60	Hepatitis	1/16,384	0	1/65,536	Acute infection (4‐fold rise)
5.	M	61	Splenomegaly, arthritis	0	1/24	1/256	Acute infection
6.	M	23	Arthritis, endocarditis, aneurysm	1/64	1/192	1/512	Acute infection
7.	M	51	Pneumonia, aneurysm, AVR	0	—	1/512	Acute infection (4‐fold rise)
8.	M	62	Aneurysm, fever, myalgia	0	—	1/512	Acute infection (4‐fold rise)
9.	F	59	Aneurysm, chronic fatigue	0	1/192	1/256	Acute infection
10.	M	73	Prosthetic valve endocarditis	0	—	1/1024	Acute infection (4‐fold rise)
11.	M	66	Pacemaker‐related endocarditis	0	1/96	1/512	Acute infection
12.	M	65	Aneurysm, vascular graft infection	1/32,768	0	1/32,768	Chronic infection

*Note:* M: male, F: female.

Abbreviation: AVR, aortic valve replacement.

**TABLE 2:** Clinical and laboratory characteristics of patients diagnosed with probable and past Q fever.

Patient	Gender	Age (years)	Clinical presentation	Phase I IgG (titer)	Phase II IgM (titer)	Phase II IgG (titer)	Diagnosis
13.	M	79	Pneumonia	0	0	1/128	Probable infection
14.	F	51	Fever, myalgia, fatigue	0	—	1/512	Probable infection (3‐fold increase)
15.	F	49	Fever, myalgia	0	0	1/256	Probable infection (2‐fold increase)
16.	F	48	Fever, myalgia	0	—	1/256	Probable infection
17.	M	79	Fever, bursitis	0	0	1/512	Probable infection
18.	M	58	Syphilis, fever, myalgia, arthralgia	0	0	1/256	Probable infection
19.	F	48	HIV, arthritis	1/128	1/96	0	Probable infection
20.	M	31	History of brucellosis myalgia, arthralgia	0	—	1/128	Probable infection (2‐fold increase)
21.	M	73	History of brucellosis myalgia, arthralgia prostatitis	1/64	—	1/2048	Probable infection
22.	M	60	Brucellosis chronic osteomyelitis	0	—	1/512	Probable infection
23.	M	47	Aortic aneurysm	0	—	1/256	Probable infection
24.	F	65	Transverse myelitis	0	—	1/128	Probable infection
25.	M	64	Fever acute renal failure	0	—	1/256	Probable infection (2‐fold increase)
26.	M	19	Myocarditis	0	0	1/512	Probable infection
27.	F	79	Endocarditis (PVE)	0	0	1/1024	Probable infection
28.	M	65	Endocarditis (PVE)	0	—	1/1024	Probable infection
29.	F	45	Necrotizing granulomatous erythema nodosum	0	0	1/64	Past infection
30.	M	48	Asymptomatic veterinarian	0	—	1/256	Past infection
31.	M	65	Cardiac tamponade lung cancer	0	0	1/64	Past infection
32.	M	74	AVR/MVR, fever	0	0	1/64	Past infection

*Note:* M: male, F: female.

Abbreviations: AVR, aortic valve replacement; HIV, human immunodeficiency virus; MVR, mitral valve replacement; PVE, prosthetic valve endocarditis.

The highest recorded serological titers were Phase II IgG at 1:65,536 and Phase I IgG at 1:32,768. In nine patients with probable infection, Phase II IgM testing could not be performed; therefore, the diagnosis could not be confirmed. The median leukocyte count was 9300/μL, CRP level was 4550 (1047–15352) nmol/L, and ESR was 53 mm/h. Additional laboratory findings are summarized in Table 3.

**TABLE 3:** Laboratory results of patients diagnosed with Q fever (*N*: 32).

Test	Result (median [25–75 IQR])
Leukocyte (10^9^/L)	9.3 (6.4–13.0)
Neutrophil (10^9^/L)	5.8 (3.7–8.3)
Lymphocyte (10^9^/L)	1.6 (1.2–2.7)
Hemoglobin (g/L)	125 (103–140)
Platelet (10^9^/L)	240 (158–287)
Aspartate aminotransferase (µkat/L)	0.41 (0.28–1.03)
Alanine aminotransferase (µkat/L)	0.42 (0.25–1.23)
Gamma‐glutamyl transferase (µkat/L)	0.72 (0.32–1.65)
Alkaline phosphatase (µkat/L)	1.67 (1.28–2.33)
C‐reactive protein (nmol/L)	4550 (1047–15352)
Sedimentation (mm/h)	53 (18–91)

## 4. Discussion

Following the increased recognition of Q fever in our region, 32 cases of *C. burnetii* infection were identified since 2023, including 11 patients diagnosed with confirmed acute infection. Although *C. burnetii* seropositivity has been demonstrated in several epidemiological surveys from Türkiye, fully documented human clinical series remain rare in the national literature [9–12]. Prior to 2023, no acute or chronic Q fever cases had been clinically reported from Ordu Province, although earlier regional seroprevalence studies had already indicated exposure among at‐risk local groups. Our findings should be interpreted as descriptive clinical observations from a selected, hospital‐based cohort rather than as direct epidemiological evidence of a rising community incidence. Ultimately, the recent cluster of recognized cases at our center most likely reflects a combination of heightened clinical awareness, expanded serological testing utilization, and improved detection of a previously underrecognized infection, rather than a point‐source outbreak or a definitive surge in community incidence, though ongoing environmental exposure pathways may also have contributed.

Q fever is capable of causing large‐scale outbreaks, as exemplified by the epidemic in the Netherlands between 2007 and 2010, during which over 4000 cases were reported [13]. In Türkiye, the first documented Q fever outbreak was reported in Aksaray in 1947 [14]. A later outbreak in Yalova in 2013 was described in a conference presentation reporting 58 cases, but detailed data from this outbreak have not been published [15]. Despite the high seroprevalence rates observed in epidemiological studies, the number of reported cases remains low, likely due to diagnostic difficulties and limited clinical awareness.

A 2023 meta‐analysis estimated the seroprevalence of *C. burnetii* in Türkiye at 22.7%. A 2017 study conducted in Ordu among high‐risk groups reported a seropositivity rate of 25.6%. Q fever is classified as a notifiable disease by inpatient healthcare institutions in Türkiye [16]. However, despite the high seroprevalence rates observed in the general population, the number of officially reported cases remains quite low. Prior to 2023, no cases of Q fever had been reported in Ordu Province, despite the presence of positivity in earlier seroprevalence studies [10]. In a 2013 study conducted in the neighboring province of Tokat, *C. burnetii* seropositivity was detected in 19 (36%) of 53 patients presenting with acute febrile illness, and 2 patients (4%) were diagnosed with acute infection [17]. Similarly, a study conducted in the Samsun region involving 407 healthy volunteers reported a seropositivity rate of 13.5%, with incidental diagnoses of acute Q fever in 17 individuals (4.1%) and chronic Q fever in 5 individuals (1.2%) [18].

Seroprevalence studies indicate that significantly more individuals have been infected with *C. burnetii* than the number of reported symptomatic Q fever cases suggests. Underreporting of Q fever may be attributed to the fact that the disease often presents with asymptomatic, subclinical, or nonspecific clinical manifestations, which can hinder accurate diagnosis. In addition, diagnosis cannot always be established with a single test; repeated testing is often required to demonstrate a fourfold increase in antibody titers. This further complicates the diagnostic process. In our study as well, for cases in which a definitive diagnosis could not be made based on the initial serological test, follow‐up serum samples were obtained at 2–4‐week intervals. Patients who demonstrated a rise in antibody titers were classified as having confirmed Q fever.

While diagnosing *C. burnetii* infection can be inherently challenging, the presence of concomitant infections introduces an added layer of complexity. In our patient cohort, coinfections such as brucellosis and HIV were identified. In addition, two patients had a documented history of brucellosis. The co‐occurrence of *Brucella spp*. and *C. burnetii* infections may be attributable to shared epidemiological risk factors, particularly among individuals with occupational or environmental exposure to livestock. Notably, a recent case report in the literature has described a spondylodiscitis involving both pathogens, further highlighting the potential for overlapping clinical presentations and the importance of considering coinfections in endemic regions [19].

Demographic factors such as age and sex have also been identified as potential risk factors for Q fever. Previous studies have consistently shown a higher prevalence of *C. burnetii* infection in males and in individuals aged between 30 and 60 years [3]. Occupational exposure, particularly among men working in agricultural or industrial settings, as well as increased cumulative exposure with advancing age, may contribute to the risk of infection. In our patient cohort, the majority were male (68.8%) and the median age was 60.5 years, aligning with previous studies that reported higher prevalence of *C. burnetii* infection among middle‐aged males.

Although leukocytosis is observed in approximately 25% of patients with acute Q fever, leukocyte counts generally remain within normal limits. Additionally, elevated ESR and CRP levels have been reported [7]. In our patient group, the median leukocyte count was 9300 cells/μL, CRP level was 4550 nmol/L, and ESR was 53 mm/h. While leukocyte counts remained within normal limits, moderate elevations were observed in acute‐phase reactants. These findings are consistent with previous studies reporting limited leukocytic response and elevated inflammatory markers in acute Q fever.

Traditionally, *C. burnetii* endocarditis has been associated with chronic Q fever; however, cases of endocarditis occurring during acute infection have also been reported. In a case series involving 759 patients with acute Q fever, valvular vegetations developed in nine individuals without any previously known valvular disease [20]. Edouard et al. reported four cases of Q fever endocarditis diagnosed by PCR or culture methods despite low Phase I antibody titers, emphasizing that low Phase I IgG levels cannot definitively exclude cardiovascular infection in patients with underlying valvulopathy [8]. This has been considered a new clinical entity possibly associated with an autoimmune mechanism during primary *C. burnetii* infection [21]. In our patient group, endocarditis was observed in three patients with Phase I antibody titers < 1:800, all of whom were diagnosed with confirmed acute infection based on positive Phase II serology. Additionally, in two patients with prosthetic valve endocarditis, the diagnosis could not be confirmed due to lack of follow‐up serological testing; thus, these cases were classified as probable infection.

Cardiovascular involvement represents one of the most serious and life‐threatening complications of Q fever. Given its association with high morbidity and mortality, recent guidelines recommend routine screening for valvular heart disease and vascular abnormalities during the acute phase, particularly in high‐risk patients [21]. Risk factors include male sex, age over 40 years, preexisting valvular disease, prosthetic valves, aneurysms, and vascular grafts [3]. In our cohort, all patients with acute infection were systematically evaluated for cardiovascular involvement using transthoracic or transesophageal echocardiography. In addition, patients were assessed for vascular abnormalities, and follow‐up continued for 1 year to detect possible progression to chronic infection. Culture‐negative endocarditis cases were also screened for Q fever. As a result, cardiovascular involvement was identified in 13 patients, which underscores the importance of systematic cardiovascular evaluation in acute Q fever cases—even when Phase I IgG titers are not markedly elevated. Although three patients with prosthetic valve endocarditis had a history of care at the same cardiac surgery clinic, this observation should be interpreted cautiously, as the available data were insufficient to establish a definite epidemiological link or confirm nosocomial transmission.

Individuals at risk for *C. burnetii* infection include livestock farmers, abattoir and laboratory workers, veterinarians, outdoor farm workers, and those exposed to contaminated manure, straw, or dust particles [22]. In this context, investigating epidemiological risk factors—such as rural residence, occupational exposure, and animal contact—is critically important in the diagnostic process. In our study, 18 patients (56.3%) were engaged in farming, 13 (40.6%) reported animal contact, and one was a practicing veterinarian. Notably, one index case had no known history of animal or farm exposure and worked in the car washing industry. Upon detailed questioning, it was revealed that the patient had fallen ill after cleaning the feces‐contaminated vehicle of a veterinarian. This underscores the critical importance of thorough epidemiological history‐taking in the diagnosis of Q fever, as indirect or covert environmental exposures can easily mask the true source of infection.

These findings should also be interpreted within a One Health framework. Recent molecular veterinary studies from Türkiye, including the Black Sea basin and the Middle and Eastern Black Sea regions, have demonstrated the circulation of *C. burnetii* in domestic ruminants and ticks, including detection of bacterial DNA in sheep and bovine aborted fetuses as well as regional tick samples. This regional veterinary evidence supports the biological plausibility of ongoing human exposure in our setting [23, 24].

The agricultural context of Ordu Province may further contribute to this exposure pattern. Ordu is one of Türkiye’s major hazelnut‐producing regions, and a substantial proportion of the population, including individuals not primarily engaged in farming, participates in seasonal hazelnut harvesting. In this context, the use of animal manure in orchards and field exposure during harvesting may create opportunities for contact with contaminated environmental material and possibly ticks, even in the absence of routine direct animal contact. Accordingly, human infection in this region may reflect not only classical livestock‐associated exposure but also seasonal and environmentally mediated exposure pathways. However, the specific contribution of hazelnut harvesting, manure use, and tick exposure to *C. burnetii* transmission in our setting remains to be clarified by further regional epidemiological studies.

This study has certain limitations. Due to its retrospective design, molecular confirmation (PCR) could not be performed on acute‐phase samples, which potentially limits diagnostic certainty for patients without complete serological follow‐up. Additionally, due to real‐world logistical constraints—including insufficient residual serum volume and temporary laboratory kit stock limitations—Phase II IgM testing could not be completed for nine probable cases. Finally, although the possibility of nosocomial transmission or clinical clustering was considered among cases infected in the same clinical setting, there was insufficient evidence to definitively confirm such transmission.

This study compiles recent clinical observations on Q fever—an infection that has long been considered rare and elusive—providing the largest single‐center clinical series from this region to date. The relatively high number of cases is one of the most remarkable findings of this study. Furthermore, the high rate of cardiovascular involvement in our patient cohort and the detection of endocarditis cases during the acute phase of infection represent important clinical observations.

## 5. Conclusions

Q fever remains a challenging disease to diagnose due to its highly variable clinical presentation and fluctuations in Phase I and Phase II antibody titers. Clinical suspicion plays a critical role in identifying cases. In Türkiye, underreporting of cases is believed to be due to low disease awareness and the complexities of the diagnostic process. Increasing awareness and improving diagnostic strategies will help detect more cases and provide better management for affected patients.

**Nomenclature**
*C. burnetii*
: *Coxiella burnetii*
LPS: Lipopolysaccharide
ELISA: Enzyme‐linked immunosorbent assay
IFA: Indirect immunofluorescence assay
AST: Aspartate aminotransferase
ALT: Alanine aminotransferase
GGT: Gamma‐glutamyl transferase
ALP: Alkaline phosphatase
CRP: C‐reactive protein
ESR: Erythrocyte sedimentation rate

## Author Contributions

Hatun Öztürk Çerik and Arzu Altunçekiç Yıldırım conceived and designed the study. Hatun Öztürk Çerik and Celali Kurt collected the data. Önder Ergönül and Hatun Öztürk Çerik analyzed the data. Hatun Öztürk Çerik wrote the manuscript.

## Funding

The authors declare that no funds, grants, or other support was received during the preparation of this manuscript.

## Disclosure

All authors read and approved the final manuscript.

## Ethics Statement

This study was performed in line with the principles of the Declaration of Helsinki. The study was approved by the Ethics Committee for Non‐Interventional Scientific Research at Ordu University (Date: April 11, 2025, Decision No: 129).

## Consent

The authors have nothing to report.

## Conflicts of Interest

The authors declare no conflicts of interest.

## Data Availability

The datasets used and/or analyzed during the current study are available from the corresponding author upon reasonable request.
